# Synthetic plant virology for nanobiotechnology and nanomedicine

**DOI:** 10.1002/wnan.1447

**Published:** 2017-01-11

**Authors:** John F. C. Steele, Hadrien Peyret, Keith Saunders, Roger Castells‐Graells, Johanna Marsian, Yulia Meshcheriakova, George P. Lomonossoff

**Affiliations:** ^1^ Department of Biology ChemistryJohn Innes CentreNorwichUK

## Abstract

Nanotechnology is a rapidly expanding field seeking to utilize nano‐scale structures for a wide range of applications. Biologically derived nanostructures, such as viruses and virus‐like particles (VLPs), provide excellent platforms for functionalization due to their physical and chemical properties. Plant viruses, and VLPs derived from them, have been used extensively in biotechnology. They have been characterized in detail over several decades and have desirable properties including high yields, robustness, and ease of purification. Through modifications to viral surfaces, either interior or exterior, plant‐virus‐derived nanoparticles have been shown to support a range of functions of potential interest to medicine and nano‐technology. In this review we highlight recent and influential achievements in the use of plant virus particles as vehicles for diverse functions: from delivery of anticancer compounds, to targeted bioimaging, vaccine production to nanowire formation. *WIREs Nanomed Nanobiotechnol* 2017, 9:e1447. doi: 10.1002/wnan.1447

For further resources related to this article, please visit the WIREs website.

## INTRODUCTION

Nanobiotechnology focusses on the use of biologically derived structures with at least one dimension smaller than 100 nm, which can be adapted to perform specific functions. In this review, we will highlight the key advancements in the use of synthetic plant virology as a basis for a number of nanobiotechnology and medical applications.

There are certain characteristics that determine the usefulness of a nanobiotechnology system. Ideally it should be possible to produce species (particles) of consistent size, structure and biophysical properties. Such particles should be amenable to the introduction of additional functional groups such as dyes, enzymes, peptides, or inorganic compounds. Also such technologies should have minimal toxicity and low impact to the environment, particularly in the case of medical applications. Additional factors that will ultimately affect the viability of a nano‐biotechnology platform include ease and cost of production, ease of containment and the low risk of cross‐contamination with mammalian pathogens.

Viruses, and noninfective virus‐like particles (VLPs) in particular, possess these desirable features. They are capable of self‐assembling into defined structures of known dimensions, show a degree of genetic flexibility to allow functionalization with proteinaceous species, and possess reactive amino acid side‐chains, that can be used for conjugation to inorganic or less amenable species. Using plant viruses reduces many of the risks associated with biological materials as a basis for medical nanotechnologies, and the use of noninfective VLPs results in low risk to the environment. These lower risks allow greater ease in handling, transportation and processing of viral nanoparticles (VNPs), making plant virus‐based particles particularly attractive platforms for a range of nanobiotechnological applications. Throughout this review, we will use VNP as a generic term to denote any particle derived primarily from viral proteins, and VLP to distinguish VNPs that do not contain viral genetic material required to be infectious.

Plant viruses have played a prominent role in biochemical and structural research, despite the chemical nature of viruses only being revealed in the mid‐1930s.^1^, ^2^ Plant viruses were an ideal system for early biochemical experiments as high viral titres, and simple purification protocols, meant high yields of reasonably pure material were easily achievable in an era preceding sophisticated recombinant technologies. These properties also led to plant viruses being in the vanguard of studies on viral architecture^3^ and were the first viruses for which detailed atomic structures were available through X‐ray crystallographic and fibre diffraction studies.^4^, ^5^, ^6^, ^7^ Concomitant with these structural studies was the determination of the nucleotide sequences of a number of plant viruses and the development of methods for the manipulation of their genomes (for a review, see Porta and Lomonossoff^8^). This solid foundation of viral research made plant viruses very attractive for exploitation as biotechnology platforms. This review seeks to introduce readers to some of the key concepts surrounding the ability to engineer plant viruses, either by genetic or chemical means, to generate synthetic nanoparticles for a wide range of applications.

### Plant Virus Structures

**Figure 1:**
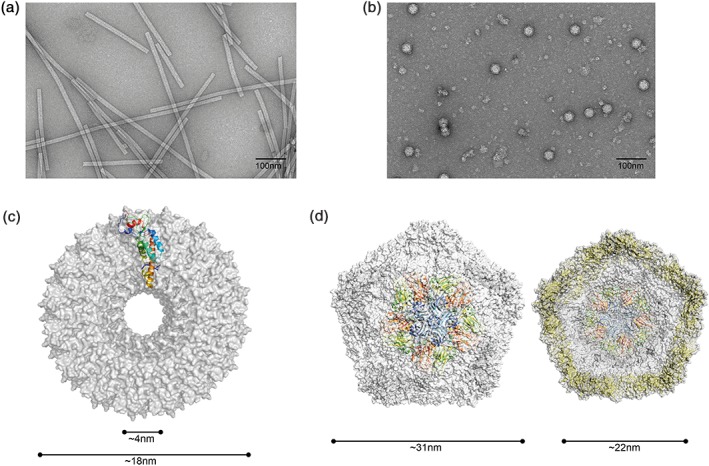
Structure and dimensions of plant viruses commonly used in nanobiotechnology. (a) Negative stain TEM of TMV showing rods of ~300 nm length. (b) Negative stain TEM of CPMV particles. (c) Cryo‐EM structure of a cross‐section of TMV (pdb 4udv). (d) Cryo‐EM structure of the external (left) and internal (right) surfaces of CPMV. Repeating motifs for cryo‐EM structures are shown as ribbons (monomer for TEM and pentamer for CPMV). [Correction added on 23 February 2017 after first online publication: labels (b) and (c) have been switched to match with the images.].

The vast majority of plant virus genera are nonenveloped and have genomes consisting of one or more strands of positive‐sense RNA. In common with all viruses, the particles are composed of highly‐repetitive motifs of protein subunits that assemble around the genome to form large, well‐ordered macromolecular structures. Though the detailed nature of these repeating motifs differs between viruses, there are essentially two classes of symmetry—helical and icosahedral^3^ (Figure 1). The classic example of a helical virus is tobacco mosaic virus (TMV, *Virgaviridae*) in which 2130 identical protein subunits surround the genome to form rigid rods of 300 nm.^4^, ^5^ Other helical viruses include potato virus X (PVX, *Alphaflexiviridae*) and potato virus Y (PVY, *Potyviridae*), both of which form flexuous rods.^9^ In terms of icosahedral symmetry, some viral capsids, such as that of Cowpea chlorotic mottle virus (CCMV, *Bromoviridae*), are comprised of multiple copies of a single‐coat protein that form icosahedral cages around its genome.^10^ Other Icosahedral viruses, such as cowpea mosaic virus (CPMV, *Secoviridae*) are composed of more than one type of capsomeric protein, though typically in this case these are produced by proteolytic processing of a precursor, meaning that the mature capsid proteins are present in equal numbers in the assembled particle.^10^, ^11^ Assembly of mature viruses typically involves formation of smaller assembly intermediates before these associate to form the large multimeric viral structures; however, the precise details of how capsid proteins mature, how assembly intermediates are initiated, and how complete structures form varies between viruses.^10^, ^12^, ^13^, ^14^, ^15^, ^16^ Regardless, the periodicity of capsid proteins in mature virions allows accurate predictions and regulation of the degree of modification that can be introduced into particles.

The precise size and highly symmetric nature of plant viruses makes them a powerful tool for structural biology, and has aided the development of structural techniques such as (cryo)‐electron microscopy. Improvements in electron microscopic techniques can be easily traced through studies into TMV structure, from development of negative stain transmission electron microscopy (TEM) methods in the late 1950s,^17^ to the advent of cryo‐EM structures defining helical arrangements 30 years later,^18^ and the rapid progression from near‐atomic resolution^19^ to atomic resolution structures that can now be produced without the need to crystallize proteins of interest.^20^ Such advances in structural biology are important for development of future biotechnological applications. Knowledge of the detailed three‐dimensional structure of virus particles resulted in identification of exposed loop regions of the coat proteins that permit genetic modification of the coat protein without interfering with the capsomeric interactions essential for assembly.^21^, ^22^

### Production of Virus and VLPs

VNPs must be produced in plants in a different way, depending on whether they are infectious viruses or noninfectious VLPs. Indeed, infectious virus can be produced by infecting plants with the relevant virus; (whether it is wild‐type or genetically modified), while VLPs must be produced by the expression of just those proteins necessary for particle formation. The latter approach has been carried out in a variety of standard heterologous expression systems including *Escherichia coli,* 23, 24 yeast^25^, ^26^, ^27^ and insect cells.^28^ The infection approach results in the production of particles containing the viral genome which may require downstream processing to remove or inactivate the viral nucleic acid^29^, ^30^for reasons of safety or containment. However, using infectious virus usually has the advantage that it is possible to produce large quantities of material as the virus is able to spread within plants. The capsid expression approach often results in the production of assembled particles *in vivo*, but unlike infectious virus, these VLPs are either devoid of nucleic acid or encapsidate host RNA. Whichever method is chosen, the assembled particles can be further processed by, for example, *in vitro* disassembly and reassembly. The capsid expression approach has the advantage that it does not require the handling of infectious material and can be used to produce mutant coat proteins that are incompatible with a ‘live’ virus infection.

Recent work in the field of synthetic plant virology has led to the development of ‘deconstructed’ viral vectors, which can be used to produce high yields of plant virus‐derived VLPs in plants rather than in a heterologous system.^31^, ^32^ The term deconstructed here refers to the removal of viral genes not required for high levels of transcription and translation (such as viral coat proteins), resulting in vectors which contain only those viral elements that lead to enhanced protein yields, such as promoters and UTRs. The first example of deconstructed viral vector use was the production of VLPs based on CPMV.^33^ Though CPMV particles produced via infection have been extensively used in bio‐ and nanotechnology (see sections below), 90% of the particles contain the viral RNA and no *in vitro* disassembly/reassembly system has yet been devised for this virus; this has limited its potential as a system for encapsidation of cargo molecules. By transiently coexpressing the precursor of the two (L and S) viral coat proteins, VP60, and the protease necessary for its processing, Saunders et al.^33^ were able to produce particles which were morphologically similar to wild‐type CPMV but which were devoid of RNA and hence were termed empty VLPs or eVLPs. Structural studies by both cryo‐EM and crystallography^34^, ^35^ revealed the particles produced in this way to be identical to wild‐type CPMV apart from the absence of the genomic RNA. Moreover, the cryo‐EM structure allowed the visualization of a 24‐amino acid chain at the C‐terminus of the S coat protein.^34^ This 24‐amino acid region plays an important role in particle assembly and controls the permeability of the particles to small molecules,^36^, ^37^ with implications for loading desired compounds into CPMV VNPs (see Section Plant Viruses as Nanocarriers of Useful Cargo below). This strategy to produce eVLPs is by no means limited to CPMV; the related virus, grapevine fanleaf virus, has only a single type of subunit corresponding to uncleaved VP60, which Belval et al. were able to express to produce RNA‐free VLPs.^38^ These eVLPs could be produced using modified coat protein that presented a marker protein on either the external or internal particle surface. Furthermore, coexpression of two different versions of the coat protein resulted mosaic particles presenting different proteins on both surfaces. Such mosaics could be of interest to medical researchers looking to deliver encapsidated compounds to specific tissues, a theme further explored in ‘VNPs for biomedical delivery and bioimaging.’

Transient expression has also been used to produce VLPs of the unrelated member of the Tombusviridae family, turnip crinkle virus^39^ (*Tombusviridae*). Expression of the full‐length coat protein led to the formation of particles closely resembling the native virus particle with 180 coat protein subunits, and these particles encapsidated host RNA. Deletion of the N‐terminal RNA‐binding domain of the coat protein resulted in the production of small RNA‐free particles containing only 60 subunits. Attempts to display foreign proteins fused to all copies of the coat protein resulted in significantly reduced yields. However, by incorporating wild‐type subunits to produce mosaic particles, this effect can be alleviated (Saunders, K., Castells‐Graells, R. and Lomonossoff, G.P., in preparation).

### *In Vitro* Assembly of Structures

The coat proteins of a number of plant viruses have been shown to be able to self‐assemble to form VLPs *in vitro*, including the well‐studied TMV.^40^ In this case, the TMV origin of assembly sequence (OAS) from the genomic RNA alone can direct particle formation by the coat protein *in vitro* under appropriate buffer conditions. By this means the assembly of rod‐shaped particles with modified coat protein conferring desirable properties to the resulting rod‐like particles has been attempted.^41^ Here, Eiben et al. note that mutant rod formation was possible only by mixing mutant coat protein synthesized in *E. coli* with wild‐type coat protein derived from a plant TMV infection in the assembly reaction. Similar *in vitro* assembly has been demonstrated for flexuous rod and icosahedral plant viruses^42^, ^43^, ^44^, ^45^ allowing the encapsidation of a range of foreign species, discussed further in Section Plant Viruses as Nanocarriers of Useful Cargo.

In addition to *in vitro* assembly to produce particles with wild‐type morphologies, filamentous viruses can be modified to produce novel structures, altering the biophysical properties of the resulting nanoparticles. Most dramatic is the production of nanospheres of various dimensions from the rod virus TMV, which maintain high thermal stability.^46^, ^47^

**Figure 2:**
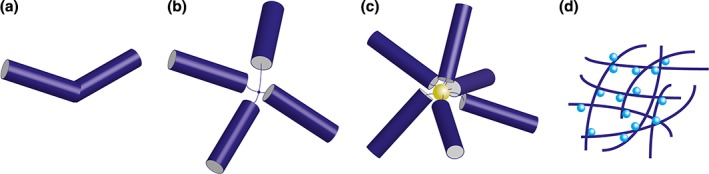
Representations of novel VNP structures for functionalization. (a) TMV‐derived ‘nano‐boomerang’ (b) TMV tetrapod, both derived by *in vitro* formation mediated by either two or four OAS on a single RNA. (c) Nano‐star formed by conjugating multiple TMV OAS to a gold nanoparticle. (d) Catalytically active TuMV nanonet formed by conjugation to *C. antarctica* Lipase B.

Of potential interest to nanobiotechnological applications, nonnative TMV structures such as ‘kinked nanoboomerangs’ can be made by introducing RNA molecules with multiple OASs, with ‘multipods’ produced in a similar manner.^48^ Such structure may allow the development of biocatalysis nanostructures with high surface area for efficient enzyme display. Metal‐nucleic acid conjugates with multiple TMV OASs results in the formation of ‘nanostars,’^48^ and similarly large networks of Turnip mosaic virus ‘Nanonets’ bound to *Candida antarctica* Lipase B resulted in catalytically active supra‐macromolecular complexes^49^ (Figure 2).

Such hybrid architectures may find favor in future nano‐technical applications such as nanowires and nanobiocatalysis assemblies. Not only can changes in nanoparticles structure alter physical properties such as surface area and thus for example reaction efficacy, but different architectures also affects *in vivo* behavior,^50^, ^51^ raising the possibility of choosing specific architectures with different pharmokinetic profiles. Although heterologous expression of TMV may be appealing for biotechnology, wild‐type TMV coat protein expressed in yeast and bacteria led to the formation of rod‐like particles of indeterminate length without the necessity of the OAS nucleotide region in the template RNA.^26^ Thus compared to *in vitro* rod formation, the heterogeneous nature of the particles formed, and lack of control over assembly normally mediated by RNA means that heterologous expression systems appears to be of limited use in this instance.

Once a particle of desired physical properties has been selected, both icosahedral and filamentous viruses structures can be functionalized via both surface modifications and use of their internal cavities.

### Functionalizing Viral Surfaces

The advent of modern recombinant technologies has allowed greater functionalization of the outer surface of viruses either by the direct genetic insertion of functional enzymes or peptides into predetermined loci, or the addition of nonnative amino acids to facilitate chemical conjugation using reactive side chains, such as those of cysteine, lysine and glutamate.

Genetic insertion of a peptide or protein may appeal to researchers with knowledge of the target virus’ structure as the position of such inserts should be predictable, and once purified the VNPs may not require additional processing for functionalization. One of the problems encountered with this approach, however, is that the presence of the inserted sequence often adversely affects the yield of virus that can be obtained, particularly if the insert is large or positively charged.^52^, ^53^ For CPMV, structural studies have been useful in rationalizing such limitations, and improving insertion sites to maximize VNP activity^22^, ^52^, ^54^; however, such data are not available for every prospective VNP. A potential solution to this problem is to use specific antibodies or antibody fragments to mediate interactions with antigens or other proteins of interest. For example, mosaic PVX VNPs have been produced *in planta*, which display engineered antibody fragments capable of antigen‐binding, despite such insertions typically preventing VNP formation.^55^, ^56^ This was achieved by including a short peptide that resulted in a mixed population of wild‐type and modified proteins via a ribosomal stutter mechanism. By choosing different antibodies for fusion to PVX coat protein in this system, it should be possible to produce VNPs functionalized with a wider range of species than would otherwise be possible.

Alternatively, researchers may choose to modify the outside of wild‐type particles by chemical conjugation in order to avoid the potentially destabilizing effect of genetic insertion. This process is not always straightforward however. For example TMV lacks exposed reactive cysteine and lysine amino acids, and so modification of wild‐type particles relies on the less commonly used azo‐coupling through existing tyrosine residues.^57^ Nevertheless, mutant TMV particles have been synthesized that include nonnative amino acids to allow thiol‐ or amine‐selective chemistry.^58^, ^59^ Similarly, modified TMV possessing a surface‐exposed cysteine residue has been coupled with sensor enzymes such as glucose oxidase and horseradish peroxidase, resulting in multivalent nanoscale platform for the ordered presentation of bioactive proteins.^60^ The scope for genetic fusions to the termini of TMV capsid proteins is limited, as this can inhibit virus formation, with wild‐type morphologies requiring mosaics of wild‐type and mutant protein.^41^

Chemical modifications to the solvent‐exposed external surface of icosahedral particle can carried out using either virus particles or VLPs, while modification of the internal surface is usually confined to empty VLPs as the presence of genomic RNA tends to occlude access to the reactive amino acid side chains.^37^ Multiple examples of surface modifications of icosahedral particles exist, using the carboxyl groups of aspartic and glutamic acid,^61^ the ε‐amino group of lysine,^62^, ^63^, ^64^, ^65^ the thiol group of cysteine^65^, ^66^ and the phenol side chain of tyrosine.^67^ It is also possible to genetically introduce or remove amino acids with reactive side changes in order to better control the levels of modification, or facilitate alternative chemistries for conjugation. For example, Wang et al.^66^ and Gillitzer et al.^68^ introduced cysteine residues to the surface of CPMV and CCMV, respectively while Chatterji et al.^69^ successively reduced the number of lysine residues on the surface of CPMV. Once a desirable chemistry has been identified (using either wild‐type or mutant VNPs), conjugation can facilitate functionalization with a wide range of species including fluorescent dyes, electroactive compounds, drug molecules, quantum dots, specific metals, and even active enzymes.^63^, ^70^, ^71^, ^72^

The interior surface of certain VNPs is also amenable to modification: the interior surface of CPMV VLPs can be chemically modified with fluorescent dyes via naturally occurring cysteines.^37^ Modification of the interior surface of VLPs derived from CCMV has also been achieved: the wild‐type coat protein is highly positively charged due to the presence of the basic amino acids lysine and arginine. This positively charged interior surface was used to promote mineralization within the preformed capsid to produce defined inorganic nanoparticles.^73^ By producing CCMV VLPs with an altered interior charge, it was possible to alter the range of materials that could be encapsulated.^74^

Modifications to both the solvent‐exposed surfaces of VLPs and to the internal cavity of many plant viruses allow synthetic viruses to interact with a variety of organic and inorganic substrates for a range of biotechnology applications, and to act as nanocarriers for both medical and bioimaging use.

### Plant Viruses as Nanocarriers of Useful Cargo

**Figure 3:**
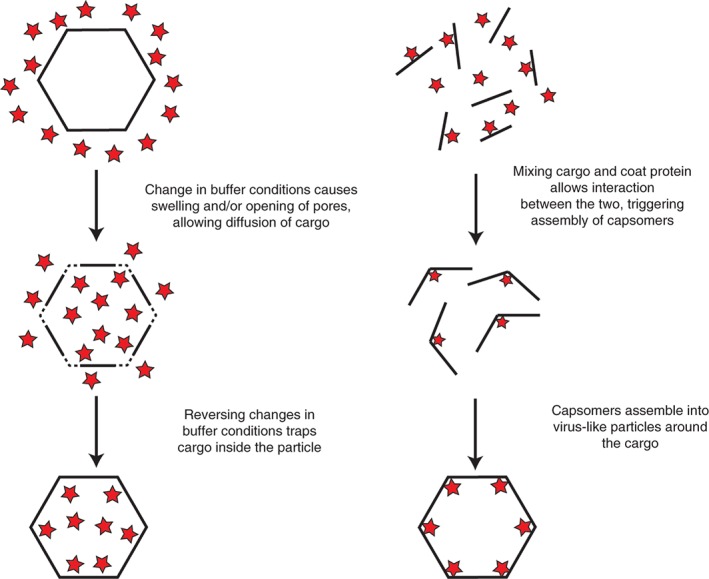
Schematic of the key methods used to encapsidate specific cargoes into VNPs. Left shows swelling‐mediated infusion of nanoparticles. Right demonstrates cargo caging.

Plant viruses have a strong track record of being used as carriers of useful cargo for a plethora of nanotechnological and biomedical applications,^75^, ^76^ which we will divide here into three broad categories: delivery of therapeutics, bioimaging, and metallization. Cargo carrying utilizes the interior viral cavity as a vehicle for specific molecules; however, the introduction of defined cargoes into viruses is nontrivial. There are two main strategies for loading plant viral particles with foreign cargo: the infusion technique seeks to allow diffusion of a cargo of interest into the preformed viral particle, whereas the caging strategy aims to trigger particle formation around the cargo of interest (Figure 3). Each strategy has been used successfully with different viruses for different types of cargo, described below.

#### Infusion

The infusion strategy relies on causing viral particles to swell in such a way that pores open in the capsid, allowing diffusion of small cargo. Reversing the swelling then causes these pores to close, thus trapping the cargo inside. The method required to achieve such reversible swelling in tomato bushy stunt virus (TBSV, *Tombusviridae*) was described by Perez et al.,^77^ and relies on the chelation then addition of divalent cations to cause opening then closing of pores. This method has been used to load TBSV virions with ethidium bromide,^78^ and a similar strategy was used by Loo et al.^79^ as well as Lockney et al.^80^ to load the chemotherapy drug doxorubicin into particles of red clover necrotic mosaic virus (RCNMV, *Tombusviridae*) thanks to the interaction between the drug and viral nucleic acid. The same technique was used by Zeng et al. to load doxorubicin into particles of the distantly related cucumber mosaic virus (CMV, *Bromoviridae*).^81^ This technique has also found a use in agronomy, where the soil mobility of RCNMV led Cao et al.^82^ to load the nematicide Abamectin into the viral particles through infusion. This encapsulated form of Abamectin had similar bioavailability to nematodes as the free form of the pesticide, but with improved soil mobility, making it more effective at protecting crop roots from nematode infection.

In some cases, infusion does not even require swelling of the viral particles to allow ingress of cargo: it is possible to use the native nucleic acid content of the virions as an electrostatic sponge to attract and retain positively charged cargo. This is the strategy that was used by Yildiz et al.^83^ as well as Wen et al.^84^ to load particles of CPMV with imaging agents and therapeutic molecules. This relies the natural affinity for these molecules with nucleic acid, and these experiments demonstrate that potential to use infused CPMV particles as imaging and therapy vehicles. A similar method was employed to load the platinum‐containing anticancer drug candidate Phenanthriplatin into the channel of the rod‐shaped TMV through a one‐step loading protocol that also exploited the electrostatic interaction between the positively charged cargo and the negatively charged interior of the viral particle.^85^

#### Caging

The caging strategy relies on assembling viral particles around the cargo of interest, either *in vivo* or after disassembling virions *in vitro*. The plant virus that has been most often used for caging of foreign cargo is almost certainly CCMV. The conditions required to disassemble the virus particles were described by Adolph,^86^ and had the advantage of being very simple: shifts in pH and ionic strength allowed for easily controlled swelling, and eventually complete disassembly of the viral particles. This mechanism has been extensively studied,^87^ and the reversibility of this process allowed later groups to use the disassembly–reassembly mechanism to remove native nucleic acid from the inside of the particles and replace it with cargo of interest. In one example, CCMV particles have been used as gene therapy candidates: the native viral RNA is removed after disassembly of the virions, and the capsid dimers are reassembled around heterologous RNA originating from the mammalian Sindbis virus.^88^ The authors even demonstrated that the heterologous RNA was released and expressed in mammalian cells upon transfection with the chimeric viral particles. Proteins can also be specifically packaged inside CCMV particles by using the caging strategy in concert with some targeted genetic engineering of the CCMV capsid protein. Indeed, the N‐terminus of the CCMV capsid protein can be fused to the K‐coil of a leucine zipper, while the C‐terminus of a protein cargo is fused to the *E. coli*, allowing noncovalent binding of the cargo to the capsid dimers, and self‐assembly of VLPs around the protein cargo.^89^

More stable covalent binding of cargo has also been induced through the use of bacterial Sortase A: The C‐terminus of the CCMV capsid protein is modified to end in a glycine residue, and covalent binding can take place with any cargo (small molecule of protein) fused to an leucine proline glutamate threonine glycine (LPETG) amino acid motif via the action of trans‐acting Sortase A.^90^ Encapsidation of whole proteins into CCMV VLPs allows the creation of nanoreactors; nanoscale cages with enzymatic activity. This was demonstrated by Comellas‐Aragones et al.,^91^ who disassembled CCMV virions and reassembled them around individual molecules of horseradish peroxidase, with the enzymatic substrate and product capable of diffusing in and out of the nanoreactors. This has huge implications for enzyme‐based therapeutics, as demonstrated by Sanchez‐Sanchez et al.,^92^ who used a similar method for the encapsulation of a bacterial cytochrome p450 inside CCMV VLPs. These VLPs were enzymatically functional and could process prodrugs into cytotoxic active forms, which has important implications for targeted drug delivery. The closely related virus CMV can also be used for caging of cargo by functionalization of a nucleic acid intermediate. Disassembled CMV capsids can be made to reassemble around heterologous DNA, which can be used directly as cargo of interest, but if the DNA is functionalized with dyes or protein (through biotin–streptavidin interactions), the capsid–DNA interaction allows encapsulation of a wide range of cargoes.^93^

Two areas which are attracting a considerable amount of attention with regards to loading cargo inside plant viral particles are the fields of biodelivery and mineralization of nano‐scale structures. To achieve their goals, researchers in both of these fields have made use of both the infusion as well as the caging strategies, each with great success.

### VNPs for Biomedical Delivery and Bioimaging

Efficient drug delivery is greatly affected by the biochemical properties of the target compound and its interactions with the host. For compounds that are quickly eliminated from the body, are poorly soluble, or may not be able to efficiently pass cell membranes, the use of carrier molecules can improve drug delivery and thus efficacy. Such carriers must be small enough to move through the bloodstream, nontoxic, biocompatible and able to enter cells. Plant viruses have all of these properties, and have been the subject of much research as potential biomedical vehicles.^94^ With distinct surface and interior residues, VNPs are good candidates for manipulation to protect poorly soluble drugs while maintaining good biocompatibility.

In addition to drug biocompatibility, efficient transport to target tissues is a major challenge in pharmacology. This is a particular issue in the case of anticancer drugs which usually discriminate between cancerous and normal cells by the fact that the cancer cells are dividing more rapidly and differentially certain cell‐surface receptors.^95^, ^96^ However, anticancer drugs are toxic to all cells and thus often have severe side effects.

Nanotechnology‐based drug delivery is aided by physiological differences between tumorous and healthy tissue. Tumors have been shown to retain more nanoparticles than healthy tissue and display increased permeability to larger nanomaterials on the basis of disrupted tight junctions. This results in preferential penetration of nanoparticles into tumorous tissue, known as the enhanced permeability and retention effect. Viruses specifically are useful for biomedical delivery due to their bioavailability, biodistribution, and persistence in a mammalian organism. Studies to ascertain these characteristics have been undertaken with CPMV,^97^, ^98^ CCMV,^99^ TMV,^100^ and PVX.^51^, ^101^, ^102^ Taken together, these studies reveal that plant viral particles are generally safe, well tolerated, with high bioavailability, broad biodistribution, and relatively short persistence in animal models.

Many wild‐type plant viruses display nonspecific cell entry, or at least entry into a wide range of cells, which may be deleterious for delivery of cytotoxic drugs. Untargeted uptake can be reduced by conjugation of surface residues to polyethylene glycol (PEGylation),^103^ and more targeted delivery facilitated by specific surface modifications. CPMV has been shown to interact with the intermediate filament protein vimentin,^104^ and this interaction facilitates CPMV uptake into a range of cells (including macrophages and certain cancerous cells). Using this natural uptake, Wen et al.^75^ were able to decorate CPMV with a photosensitizing agent to target macrophages and tumorous cells for destruction. Similarly, the loading of TMV with Zn‐EpPor has been shown to enhance photosensitizer uptake into melanoma cells.^105^

Integrins are another example of cell‐surface receptors that are upregulated in many cancers.^106^ Specific peptide motifs, such as the RGD motif seen in Adenovirus are recognized by subtypes of integrins, and when this peptide is introduced to the surface of CPMV, either by genetic fusion to exposed loops or via chemical conjugation, uptake of CPMV into multiple cancer cell lines can be increased.^107^ Similar approaches have been used to target ovarian cancer cells. Folic acid receptors (FR) are overexpressed in ovarian cancer cells, and so FR‐mediated endocytosis is a potential strategy for cell‐specific drug delivery.^108^ This strategy was successfully employed by Ren et al.^109^ and Zeng et al.^81^: modification of the surfaces of two unrelated viruses with folic acid for cell targeting, and infusion of the internal cavity of the VNPs with doxorubicin resulted in a significant increase in drug delivery into cancer cells.

The ability to incorporate custom cargoes into protective protein shells, and targeting of these to specific tissue types allows the use of VNPs as bioimaging agents. Using NHS ester chemistry both flexuous rod virus PVX and the icosahedral virus CPMV can be functionalized with a number of commercial fluorescent dyes for imaging in cell cultures and to mark embryo vasculature and tumor tissue in chick and mouse systems.^37^, ^110^, ^111^, ^112^, ^113^ The use of more complex two‐step bioconjugation methods allows further functionalization of PVX to target fluorescent particle uptake into cancer cells.^114^ In addition to commonly used single‐photon fluorescent markers, two‐photon dyes can be preferable for bioimaging as such dyes give lower background fluorescence^115^ and can reduce radiation damage to surrounding tissue.^116^, ^117^ Recently the coupling of TMV particles to the two‐photon dye BF_3_‐NCS has been demonstrated to allow visualization of diseased brain vasculature in mice.^118^ Imaging using nonoptical methods such as MRI is also achievable by loading metals such as gadolinium into VNPs.^119^, ^120^ Such metal‐VNP‐specific interactions, referred to as mineralization, are further discussed in Section Mineralization of Viral Scaffolds.

VNPs provide a good basis for biomedical technology, not only as chassis capable of transporting specific cargo, but also as scaffolds for antigen presentation and vaccination. With the development of plant‐virus based technologies capable of producing a wide range of non‐infectious VLPs, plant‐derived VLPs is an area of considerable interest.

### Synthetic Plant Virology for Vaccine Production

Vaccines provide acquired immunity to the infection of a particular micro‐organism by stimulating the immune system against it. Vaccines against viral diseases are commonly produced from lab‐cultured pathogens which are then attenuated or inactivated. This comes with the risk of residual pathogenic activity due to incomplete inactivation, attenuation, or reversion.^121^ A potentially safer approach is therefore the recombinant production of specific components of pathogens, which can mimic the immunological properties of the original virus without its pathogenic properties.

Heterologous production and purification of immunogenic regions, for example solvent‐exposed surface peptides, that can be introduced into hosts can result in immune responses. The use of nanoparticles such as VLPs as display scaffolds, enhances the immunogenicity of the antigen by presenting a quasi‐crystalline ordered repeat antigen structure to the immune system^122^, ^123^ (further explored in Plummer and Manchester^124^). Because the point of an antigen‐carrier VNP is that the VNP moiety is incapable of causing an infection in the animal host, replicating plant viruses used for antigen display can be considered antigen‐carrier VLPs, even though they are not technically VLPs. There are numerous examples of replicating plant viruses used to display immunogenic epitopes of animal pathogens for vaccine purposes. Chimaeric CPMV particles displaying a foot and mouth disease virus epitope, human rhinovirus epitope, or HIV epitope fused to the S coat protein have been produced in cowpea plants, and these particles could stimulate an immune response against the target epitope in test animals.^125^, ^126^, ^127^ Moreover, this technique also permitted the development of a vaccine against mink enteritis virus that provides protective immunity in target animals.^128^ TMV particles have also been used to display heterologous epitopes: a leaky stop codon strategy was used to produce chimaeric TMV particles in which the C‐terminus of some of the coat proteins is fused to epitopes from the malaria parasite,^129^ influenza virus, or HIV.^130^ It has subsequently shown that TMV particles presenting a short epitope from murine hepatitis virus was able to stimulate protective immunity in mice.^131^

Chemical conjugation of antigens provides a further alternative to genetic fusion, with tyrosine‐mediated linkage of the weakly immunogenic hapten estriol to the surface of TMV leading to a significant immune response in mice.^132^

Although VLP‐mediated antigen display can increase immune protection, this is not always the case.^133^, ^134^, ^135^ Furthermore, the use of a single VLP scaffold for antigen delivery may not be sustainable as exposure to the scaffold may lead to an immune response against the VLP moiety. Instead, recent work in the field of synthetic plant virology has used deconstructed viral vectors to produce high yields of pharmaceutically interesting proteins (for a recent review, see Peyret & Lomonossoff^32^).

Although beyond the scope of this review to cover in detail, the use of such vectors and heterologous plant systems such as *Nicotiana benthamiana* to produce nonplant VLPs for vaccines is an exciting area of research that cannot go without mention. In this approach the structural proteins for a target virus are cloned into a high‐expression vector and transiently expressed in hosts such as *N. benthamiana*. Protein expression results in particles that morphologically resemble their infectious counterparts, and thus can be expected to act as an effective vaccine. Similar methods can be used in traditional mammalian and insect cell‐culture; however, the use of plants has a number of significant benefits. This approach has been used successfully to produce a number of different VLPs as vaccine candidates (Table 1). It is possible to produce VLPs composed of a small number of capsid proteins, such as Hepatitis B core‐like particles and Human Papillomavirus VLPs,^142^, ^149^ and more complex nonenveloped proteins such as Bluetongue virus.^148^ The transient expression in plants of VLPs from a variety of animal viruses is further reviewed in Marsian and Lomonossoff, 2016.^150^

**Table 1:** Key Vaccine Candidates Produced Using Deconstructed Viral Vectors. Further Details Can Be Found in Marsian and Lomonossoff 2016.

Virus	Summary	Reference
Hepatitis B	Tabletized transgenic lettuce containing HBsAg VLPs is orally immunogenic in mice	Pniewski 2011^136^
Hepatitis C	Cucumber mosaic virus nanoparticles carrying a Hepatitis C virus‐derived epitope, orally immunogenic in rabbits	Nuzzaci 2010^137^
Hepatitis C	Papaya mosaic virus‐like particles fused to a hepatitis C virus epitope: evidence for the critical function of multimerization, mixed response in mice	Denis 2007^138^
Influenza	Influenza virus‐like particles induce a protective immune response against a lethal viral challenge in mice, produced for H7N9 outbreak virus	D'Aoust 2008^139^
Papillomavirus	HPV‐16 L1 VLPs via agroinfiltration‐mediated transient expression or via transplastomic expression	Maclean 2007,^140^ Fernandez‐San 2008^141^
Papillomavirus	Expression of HPV‐8 L1 VLPs	Matic 2012^142^
Papillomavirus	transient expression of chimaeric L1::L2 VLPs and proof of increased breadth of immune response	Pineo 2013^143^
Bovine papillomavirus	Transient expression of BPV L1 VLPs	Love 2012^144^
HIV	Expression of Gag VLPs in transgenic tobacco chloroplasts	Scotti 2009^145^
Human norovirus	NaVCP VLPs in which generate a mucosal and serum antibody response	Mathew 2014^146^
Rotavirus	Immunogenic rotavirus‐like particles in transgenic plants	Yang 2011^147^
Bluetongue virus	Protective bluetongue virus‐like particles	Thuenemann 2013^148^

### Mineralization of Viral Scaffolds

**Figure 4:**
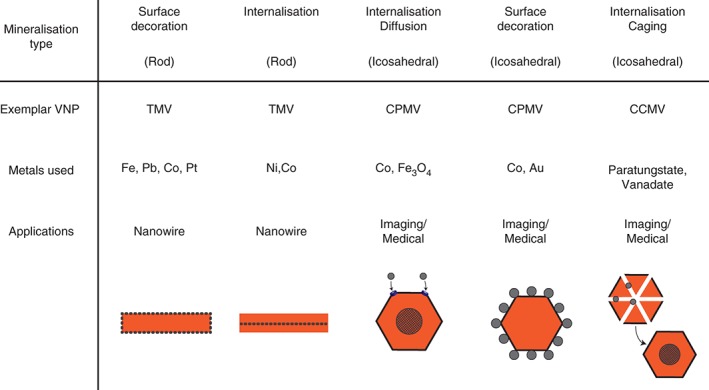
Summary of different VNP‐metal interactions. Both icosahedral and rod‐shaped viruses can be used to either cage metals, or act as scaffolds to decorate with a variety of metals for a range of biomedical and nano‐biotechnological functions.

Many VNP technological and medical applications, such MRI, are dependent on the ability to form complexes between VNPs and specific metals and metal‐based compounds. In the case of mineralization, inorganic nanoparticles and microstructures can be deposited and assembled on virus structures that act as biological templates or scaffolds.^151^, ^152^ Mineralization has been reported for both the interior and exterior surfaces of icosahedral and rod‐shaped virus particles (Figure 4). VNPs present several advantages like their nanoscale size, symmetry, polyvalence and monodispersity.^75^, ^152^ Hybrid organic–inorganic species such as metallized VNPs can be useful for a broad range of applications such as catalysis, semiconductors, drugs and contrast agents.^73^, ^75^

Icosahedral viruses such as CCMV and CPMV have been demonstrated to be able to interact with a variety of metals, with the positively charged internal cavity of CCMV allowing inorganic crystal nucleation.^153^ pH‐dependent swelling, as described above, results in the formation of 60 pores in the capsid, which can then be used to load paratungstate and vanadate.^73^ CPMV shows similarities to CCMV in its ability be loaded with metals cobalt or iron‐oxide^154^; however, in order to load CCMV with iron, nine basic amino acids at the N‐terminus of the coat protein must be substituted with glutamate residues to alter the charge of the inner cavity to allow oxidative mineralization.^74^, ^155^ The surface of CPMV can be decorated directly with ferrocene derivatives.^154^ Alternatively, custom decoration with cobalt–platinum, iron–platinum or zinc sulfide can be achieved by conjugating mineralizing peptides to the viral surface, or genetic fusion of hexa‐histidine motifs to the C‐terminus of the small coat protein subunit.^36^, ^156^

Filamentous viruses such as TMV can also be modified to facilitate mineralization, either externally or internally. Immobilization of both silica and a range of metals on the surface of TMV has been demonstrated,^151^, ^152^, ^157^, ^158^ and the inner cavity can likewise be utilized for mineralization of nickel and cobalt.^159^, ^160^ With a range of available VNPs with different physical characteristics (icosahedral, rigid, or flexuous rod), and by identifying the appropriate modifications required for specific metal interactions, VNPs appear to be a viable platform for the generation of a number of organic–inorganic hybrids.

## CONCLUSION

Plant viruses are an integral tool for nanobiotechnology. Much of this is due to the extensive foundational work regarding plant virus structure, genetics, and biochemistry. Viruses have many properties making them ideal chassis for biotechnological functions, with plant viruses having additional benefits with regards to ease of production and purification. Production is one of the main limiting factors for widespread use of plant‐derived VNPs, as current research can be relatively labor‐intensive; activities such as pricking out seedlings for growth are typically done manually. This issue is by no means insurmountable, as automation is feasible for most aspects of plant growth, and such issues must be weighed against the benefits of using simple media and growth conditions. The success of companies such as North America‐based Medicago using plants as an expression system demonstrate the viability of industrial‐scale use of plants for protein expression.

As both wild‐type viruses and noninfectious VLPs pose little threat to human health, they are a good choice for nanomedical uses. As with any protein‐based system there are risks of inducing an immune response to VNPs which may limit their long‐term usefulness in both antigen display and live‐animal bioimaging; however, to what extent this will prevent their clinical usefulness is uncertain. With very promising preclinical data regarding VNP toxicity, retention, and biodistribution, a key area of research will be the demonstration of clinical efficacy. If VNPs can be demonstrated to be viable clinically, improved methods for cell targeting will be crucial for widespread use of VNPs for drug delivery and gene therapy. The development of resources such as TumorHOPE,^161^ a database for tumor homing peptides, will play a vital role in developing novel targeting methods that may be ultimately beneficial where existing drug treatments show little discrimination between diseased and healthy tissue.

Functionalization of external surfaces combined with manipulation of viral morphologies opens a large number of potential applications in nanotechnology, from nanowires to nanoreactors. Increasing our understanding of virus tolerance to manipulation, generation of more novel structures, and methods of functionalizing with active enzymes without affecting activity is going to be key in expanding the range of VNPs with custom properties.

Currently there are no plant‐based nanobiotechnology products on the market. We believe that as researchers are able to demonstrate greater success in producing VNPs with desirable properties, increased interest from both private and public bodies will drive investment in both fundamental research and infrastructure required to produce economically viable technologies. Part of this is likely to be due to economies of scale: despite low running costs for plant production, high upfront costs for industrial‐scale expression means that very few sites are capable of producing industrial levels of plant‐derived nanotech materials. Specialized medium‐scale production facilities that will allow researchers to test the feasibility of novel VNPs will be an important step in demonstrating viability of plants as an expression platform, thus hopefully stimulating wider interest in the field.

Furthermore, new technologies are likely to be a driving force in plant‐based VNP production, and plant‐based heterologous expression in general. Cell‐based methods such as the BY2 cell‐pack method for transient expression^162^ that allow medium‐ to high‐throughput screening will be essential to allow candidate screening analogous to existing *E. coli* based methods. Although the production timescales are unlikely to match those of *E. coli* (expression typically requiring several days as opposed to several hours), transient expression screens are still a viable means to facilitate construct design and optimization for enhanced yield, and improved particle characteristics.
